# Supplementary material for: High SARS-CoV-2 incidence and asymptomatic fraction during Delta and Omicron BA.1 waves in The Gambia
Source: Nat Commun. 2024 May 7;15:3814. doi: 10.1038/s41467-024-48098-3 (PMC11076623; doi:10.1038/s41467-024-48098-3)
Supplement: Supplementary file 1 — Supplementary Information [file 41467_2024_48098_MOESM1_ESM.docx]

**Supplementary Appendix**

Contents

[*Table S1 – The association between prior infection status (including time since last infection) and the incidence of infection.* 2](#_Toc160951467)

[*Table S2 – A summary of individual-level SARS-CoV-2 infection data for each infection scenario* 3](#_Toc160951468)

[*Table S3 – The association between prior infection status, age, household size and SARS-CoV-2 incidence for each infection scenario* 4](#_Toc160951469)

[*Table S4 – The effect of variant on the association between prior infection/age and SARS-CoV-2 incidence for each infection scenario.* 6](#_Toc160951470)

[*Table S5 – The frequency of SARS-CoV-2 symptoms reported by those with symptomatic infections*. 7](#_Toc160951471)

[*Table S6 – The univariate association between prior infection status, variant, age, household size and demographic/clinical variables with the odds of an infection being symptomatic* 8](#_Toc160951472)

[*Table S7 – The univariate and multivariate associations between prior infection status, variant, and age with the odds of an infection being symptomatic* 9](#_Toc160951473)

[*Table S8 – Association between index participant factors and household factors and the odds of secondary transmission of SARS-CoV-2 in a single-index cluster (Secondary Attack Rate).* 10](#_Toc160951474)

[*Table S9 – Multivariate association between index SARS-CoV-2 serology and the odds of subsequent transmission of SARS-CoV-2 during a cluster after exposure to a single index (Secondary Attack Rate).* 11](#_Toc160951475)

[*Table S10 – Association between index participant factors and household factors and the odds of subsequent transmission of SARS-CoV-2 during a cluster after exposure to a single index (Household Cumulative Infection Risk).* 12](#_Toc160951476)

[*Table S11 – Multivariate association between index SARS-CoV-2 serology and the odds of subsequent transmission of SARS-CoV-2 during a cluster after exposure to a single index (Household Cumulative Infection Risk).* 13](#_Toc160951477)

[*Table S12 – Summary of PCR-negative scenarios for sensitivity analyses* 14](#_Toc160951478)

[*Determining the seroconversion threshold through mixture modelling* 15](#_Toc160951479)

[*Figure S1 – Imputed dates of PCR-negative infections for scenarios B, and 1-7. For each scenario the imputed PCR-negative infections dates (bottom plot) are compared against the epidemic curve of The Gambia during TransVir follow-up (top plot)* 16](#_Toc160951480)

***Table S1. The association between prior infection status (including time since last infection) and the incidence of infection.***

| **Variable** | **Level** | **Adjusted HR (95% CI)*** | **p-value**** | **Delta-specific aHR (95% CI)***** | **Omicron-specific aHR (95% CI)***** | **p-value**  **for interaction** |
| --- | --- | --- | --- | --- | --- | --- |
| **Prior Infection** | No prior infection | ref |  | ref |  |  |
|  | 1 prior infection < 90 days | 0.44 (0.24 – 0.79) |  | 0.29 (0.12– 0.67) | 0.73 (0.28 – 1.91) |  |
|  | 1 prior infection ≥ 90 days | 0.42 (0.32 – 0.58) |  | 0.28 (0.19– 0.43) | 0.75 (0.50 – 1.14) |  |
|  | 2 or more prior infections < 90 days | 0.15 (0.08 – 0.29) |  | 0.09 (0.02– 0.38) | 0.23 (0.11 – 0.47) |  |
|  | 2 or more prior infections ≥ 90 days | 0.13 (0.08 – 0.19) | <0.0001 | 0.03 (0.01 -0.14) | 0.23 (0.14 – 0.37) | <0.0001 |

*All models use the Anderson-Gill extension of the proportional cox hazards model, accounting for clustering by participant and household. *Adjusted for prior infection status, age, household size and vaccination status stratified by period. **P-values were calculated through likelihood ratio tests of nested models and are two-sided. ***Calculated through the incorporation with an interaction term between the variable of interest and period with adjustment for prior infection status, age, household size and vaccination status. P-values for interaction and were calculated through likelihood ratio tests of models with and without interaction terms.*

| **Scenario** | **Number of participants infected (%)*** | **Number of PCR-negative infections** | **Number of infections (PCR+ and PCR-)** | **1st infection during follow-up** | **2nd infection during follow-up** | **3rd infection during follow-up** | **Number of infections pre-delta** | **Number of infections during delta** | **Number of infections during Omicron** | **Infection Risk Pre-delta %**** | **Infection Risk Delta %**** | **Infection Risk Omicron %**** |
| --- | --- | --- | --- | --- | --- | --- | --- | --- | --- | --- | --- | --- |
| Base | 270 (79.9) | 111 | 381 | 270 | 99 | 12 | 40 | 148 | 177 | 11.8 | 44.6 | 56.7 |
| Scenario 1 | 284 (84.0) | 161 | 430 | 284 | 133 | 13 | 39 | 175 | 198 | 11.5 | 52.7 | 63.5 |
| Scenario 2 | 285 (84.3) | 161 | 430 | 284 | 133 | 13 | 44 | 171 | 197 | 13 | 51.5 | 63.1 |
| Scenario 3 | 275 (81.4) | 124 | 394 | 275 | 107 | 12 | 37 | 157 | 183 | 10.9 | 47.3 | 58.7 |
| Scenario 4 | 277 (82.0) | 129 | 398 | 277 | 109 | 12 | 35 | 165 | 180 | 10.3 | 49.7 | 57.7 |
| Scenario 5 | 270 (79.9) | 110 | 380 | 270 | 98 | 12 | 34 | 154 | 176 | 10 | 46.4 | 56.4 |
| Scenario 6 | 269 (79.6) | 133 | 403 | 269 | 121 | 13 | 39 | 161 | 185 | 11.5 | 48.5 | 59.3 |
| Scenario 7 | 269 (79.6) | 108 | 356 | 269 | 84 | 3 | 37 | 150 | 167 | 10.9 | 45.2 | 53.5 |
| Scenario 8 | 204 (60.4) | 0 | 270 | 204 | 54 | 12 | 31 | 109 | 114 | 9.1 | 32.8 | 36.5 |

***Table S2. A summary of individual-level SARS-CoV-2 infection data for each infection scenario.***

**% indicates the proportion of all participants (N=338 that completed at least 5 follow-up visits) that were infected. **calculated as number of participants infected during each variant period / number of participants who had at-least 1 follow-up visit per variant period.*

***Table S3. The association between prior infection status, age, household size and SARS-CoV-2 incidence for each infection scenario.***

|  |  | **Base Scenario** | | | | **Scenario 1 (IQR1SN + MedianNV)** | | | | **Scenario 2 (IQR1SN + IQR1NV)** | | | |
| --- | --- | --- | --- | --- | --- | --- | --- | --- | --- | --- | --- | --- | --- |
| **Variable** | **Level** | **Crude HR (CI)** | **p** | **Adjusted HR (CI)*** | **p** | **Crude HR (CI)** | **p** | **Adjusted HR (CI)*** | **p** | **Crude HR (CI)** | **p** | **Adjusted HR (CI)*** | **p** |
| **Age (years)** | <5 | 0.80 (0.60-1.07) |  | 0.48 (0.31-0.74) |  | 0.84 (0.65-1.08) | 0.74 | 0.52 (0.34-0.78) |  | 0.83 (0.64-1.07) | | 0.52 (0.34-0.79) |  |
|  | 5-17 | 1.00 (0.86-1.17) |  | 0.84 (0.64-1.10) |  | 0.99 (0.86-1.13) | 0.74 | 0.77 (0.61-0.97) |  | 0.99 (0.86-1.13) | | 0.79 (0.63-1.00) |  |
|  | 18-49 | Ref |  | ref |  | Ref |  | ref |  | ref |  | ref |  |
|  | ≥50 | 1.08 (0.83-1.40) | 0.61 | 1.07 (0.72-1.59) | 0.0002 | 1.05 (0.85-1.30) | 0.74 | 1.09 (0.79-1.49) | 0.001 | 1.08 (0.88-1.32) | 0.66 | 1.1 (0.82-1.48) | 0.002 |
| **Prior Infection** | 0 | ref |  | ref |  | ref |  | ref |  | ref |  | ref |  |
|  | 1 | 0.70 (0.58-0.84) |  | 0.42 (0.32-0.56) |  | 0.84 (0.70-1.00) |  | 0.57 (0.43-0.75) |  | 0.86 (0.71-1.03) | | 0.57 (0.43-0.76) |  |
|  | ≥2 | 0.38 (0.29-0.51) | <0.0001 | 0.13 (0.09-0.20) | <0.0001 | 0.48 (0.38-0.61) | <0.0001 | 0.18 (0.13-0.26) | <0.0001 | 0.5 (0.40-0.63) | <0.0001 | 0.19 (0.13-0.26) | <0.0001 |
| **Household Size** | 5-7 | Ref |  | ref |  | ref |  | ref |  | ref |  | ref |  |
|  | 8-10 | 0.93 (0.79-1.09) |  | 1.06 (0.83-1.36) | 0.87 | 0.98 (0.86-1.13) | 0.97 | 1.05 (0.84-1.30) |  | 0.98 (0.85-1.13) | | 1.04 (0.84-1.30) |  |
|  | >10 | 0.98 (0.82-1.17) | 0.81 | 1.05 (0.78-1.41) | 0.87 | 0.97 (0.82-1.14) | 0.97 | 0.97 (0.74-1.26) | 0.82 | 0.98 (0.83-1.15) | 0.98 | 0.99 (0.76-1.29) | 0.89 |
|  |  | **Scenario 3 (MedianS + MedianNV)** | | | | **Scenario 4 (MedianN + MedianNV)** | | | | **Scenario 5 (MixtureS + MixtureNV)** | | | |
|  |  | **Crude HR (CI)** | **p** | **Adjusted HR (CI)*** | **p** | **Crude HR (CI)** | **p** | **Adjusted HR (CI)*** | **p** | **Crude HR (CI)** | **p** | **Adjusted HR (CI)*** | **p** |
| **Age (years)** | <5 | 0.84 (0.64-1.10) |  | 0.51 (0.34-0.78) |  | 0.80 (0.60-1.05) |  | 0.49 (0.32-0.75) |  | 0.78 (0.58-1.06) | | 0.46 (0.30-0.72) |  |
|  | 5-17 | 1.02 (0.88-1.19) |  | 0.83 (0.64-1.08) |  | 0.96 (0.84-1.11) |  | 0.77 (0.60-0.99) |  | 1.00 (0.85-1.16) |  | 0.8 (0.62-1.05) |  |
|  | 18-49 | ref |  | ref |  | ref |  | ref |  | ref |  | ref |  |
|  | ≥50 | 1.15 (0.92-1.43) | 0.59 | 1.20 (0.88-1.65) | 0.003 | 1.00 (0.77-1.28) | 0.65 | 0.91 (0.63-1.31) | 0.002 | 1.21 (0.97-1.51) | 0.35 | 1.22 (0.88-1.69) | 0.0004 |
| **Prior Infection** | 0 | ref |  | ref |  | ref |  | ref |  | ref |  | ref |  |
|  | 1 | 0.75 (0.63-0.90) |  | 0.47 (0.35-0.64) |  | 0.76 (0.64-0.91) |  | 0.47 (0.36-0.63) |  | 0.7 (0.58-0.84) | | 0.42 (0.31-0.56) |  |
|  | ≥2 | 0.40 (0.30-0.52) | <0.0001 | 0.14 (0.09-0.21) | <0.0001 | 0.42 (0.33-0.55) | <0.0001 | 0.14 (0.10-0.21) | <0.0001 | 0.39 (0.30-0.52) | <0.0001 | 0.13 (0.09-0.20) | <0.0001 |
| **Household Size** | 5-7 | ref |  | ref |  | ref |  | ref |  | ref |  | ref |  |
|  | 8-10 | 0.95 (0.82-1.11) |  | 1.09 (0.86-1.38) |  | 0.93 (0.80-1.08) |  | 0.99 (0.78-1.24) |  | 0.94 (0.80-1.10) | | 1.09 (0.86-1.39) |  |
|  | >10 | 1.00 (0.84-1.19) | 0.89 | 1.05 (0.79-1.40) | 0.8 | 0.97 (0.82-1.15) | 0.82 | 0.99 (0.75-1.29) | 0.99 | 0.98 (0.82-1.17) | 0.88 | 1.04 (0.77-1.40) | 0.78 |
|  |  | **Scenario 6 (MixtureN + MixtureNV)** | | | | **Scenario 7 (90 days + Base)** | | | | **Scenario 8 (PCR+ only)** | | | |
|  |  | **Crude HR (CI)** | **p** | **Adjusted HR (CI)*** | **p** | **Crude HR (CI)** | **p** | **Adjusted HR (CI)*** | **p** | **Crude HR (CI)** | **p** | **Adjusted HR (CI)*** | **p** |
| **Age (years)** | <5 | 0.82 (0.63-1.08) |  | 0.51 (0.34-0.78) |  | 0.68 (0.49-0.93) |  | 0.41 (0.27-0.63) |  | 0.93 (0.61-1.42) | | 0.60 (0.36-1.02) |  |
|  | 5-17 | 0.98 (0.84-1.14) |  | 0.79 (0.62-1.01) |  | 0.98 (0.82-1.17) |  | 0.79 (0.61-1.03) |  | 1.08 (0.85-1.38) | | 0.87 (0.64-1.17) |  |
|  | 18-49 | ref |  | ref |  | ref |  | ref |  | ref |  | ref |  |
|  | ≥50 | 1.18 (0.93-1.51) | 0.49 | 1.22 (0.85-1.75) | 0.001 | 0.99 (0.73-1.34) | 0.22 | 0.9 (0.62-1.33) | 0.0003 | 1.36 (0.94-1.98) | 0.52 | 1.47 (0.95-2.28) | 0.03 |
| **Prior Infection** | 0 | ref |  | ref |  | ref |  | ref |  | ref |  | ref |  |
|  | 1 | 0.75 (0.63-0.91) |  | 0.48 (0.37-0.64) |  | 0.76 (0.63-0.90) |  | 0.45 (0.34-0.60) |  | 0.63 (0.49-0.80) | | 0.46 (0.33-0.63) |  |
|  | ≥2 | 0.46 (0.36-0.59) | <0.0001 | 0.17 (0.12-0.24) | <0.0001 | 0.57 (0.41-0.79) | 0.004 | 0.19 (0.12-0.28) | <0.0001 | 0.56 (0.37-0.83) | 0.001 | 0.28 (0.18-0.45) | <0.0001 |
| **Household Size** | 5-7 | ref |  | ref |  | ref |  | ref |  | ref |  | ref |  |
|  | 8-10 | 0.90 (0.77-1.05) |  | 0.95 (0.75-1.19) |  | 0.91 (0.76-1.11) |  | 0.96 (0.75-1.23) |  | 0.87 (0.68-1.11) | | 0.89 (0.67-1.18) |  |
|  | >10 | 0.92 (0.77-1.10) | 0.64 | 0.90 (0.69-1.18) | 0.72 | 0.96 (0.78-1.19) | 0.73 | 0.99 (0.75-1.32) | 0.939 | 0.97 (0.74-1.27) | 0.58 | 1.00 (0.72-1.38) | 0.65 |

*P-values were calculated through likelihood ratio tests of nested models and are two-sided. All models use the Anderson-Gill extension of the proportional cox hazards model, accounting for clustering by participant and household. Number of infections defined as per scenarios in Table S1. *Adjusted for prior infection status, age, household size and vaccination status stratified by period.*

***Table S4. The effect of variant on the association between prior infection/age and SARS-CoV-2 incidence for each infection scenario.***

|  |  | **Base (MedianSN + MedianNV)** | | | **Scenario 1 (IQRS1N + NV)** | | | **Scenario 2 (IQR1SN + IQR1NV)** | | |
| --- | --- | --- | --- | --- | --- | --- | --- | --- | --- | --- |
| **Variable** | **Level** | **Delta aHR* (CI)** | **Omicron aHR* (CI)** | **p-value** | **Delta aHR* (CI)** | **Omicron aHR* (CI)** | **p-value** | **Delta aHR* (CI)** | **Omicron aHR* (CI)** | **p-value** |
| **Age (years)** | <5 | 0.54 (0.30-0.98) | 0.43 (0.25-0.74) |  | 0.59 (0.35-1.00) | 0.48 (0.29-0.79) |  | 0.63 (0.37-1.05) | 0.45 (0.27-0.75) |  |
|  | 5-17 | 0.93 (0.64-1.36) | 0.75 (0.54-1.03) |  | 0.95 (0.68-1.32) | 0.68 (0.51-0.90) |  | 0.94 (0.67-1.32) | 0.69 (0.52-0.92) |  |
|  | 18-49 | ref | ref |  | Ref | ref |  | Ref | Ref |  |
|  | ≥50 | 1.31 (0.71-2.41) | 0.86 (0.51-1.46) | 0.64 | 1.23 (0.68-2.21) | 0.96 (0.65-1.32) | 0.55 | 1.44 (0.88-2.38) | 0.88 (0.57-1.35) | 0.77 |
| **Prior Infections** | 0 | ref | ref |  | Ref | ref |  | ref | ref |  |
|  | 1 | 0.28 (0.18-0.42) | 0.74 (0.49-1.13) |  | 0.45 (0.320-0.67) | 0.87 (0.58-1.32) |  | 0.42 (0.29-0.63) | 0.88 (0.57-1.36) |  |
|  | ≥2 | 0.05 (0.02-0.14) | 0.23 (0.14-0.36) | 0.0002 | 0.06 (0.02-0.15) | 0.33 (0.22-0.50) | 0.0004 | 0.04 (0.01-0.13) | 0.33 (0.22-0.52) | 0.0003 |
|  |  | **Scenario 3 (MedianS + MedianNV)** | | | **Scenario 4 (MedianN + MedianNV)** | | | **Scenario 5 (MixtureS + MixtureNV)** | | |
|  | **Level** | **Delta aHR* (CI)** | **Omicron aHR* (CI)** | **p-value** | **Delta aHR* (CI)** | **Omicron aHR* (CI)** | **p-value** | **Delta aHR* (CI)** | **Omicron aHR* (CI)** | **p-value** |
| **Age (years)** | <5 | 0.51 (0.29-0.91) | 0.50 (0.30-0.83) |  | 0.55 (0.31-0.99) | 0.42 (0.25-0.72) |  | 0.47 (0.26-0.86) | 0.43 (0.25-0.73) |  |
|  | 5-17 | 0.92 (0.64-1.32) | 0.75 (0.55-1.02) |  | 0.91 (0.63-1.32) | 0.68 (0.50-0.93) |  | 0.88 (0.60-1.28) | 0.68 (0.50-0.93) |  |
|  | 18-49 | ref | ref |  | ref | ref |  | ref | ref |  |
|  | ≥50 | 1.25 (0.67-2.31) | 1.15 (0.78-1.67) | 0.86 | 1.14 (0.61-2.11) | 0.77 (0.46-1.29) | 0.79 | 1.65 (0.94-2.91) | 1.13 (0.72-1.76) | 0.83 |
| **Prior Infections** | 0 | ref | ref |  | ref | ref |  | ref | ref |  |
|  | 1 | 0.32 (0.21-0.48) | 0.77 (0.50-1.19) |  | 0.32 (0.21-0.48) | 0.79 (0.52-1.21) |  | 0.28 (0.19-0.42) | 0.71 (0.47-1.07) |  |
|  | ≥2 | 0.05 (0.02-0.13) | 0.23 (0.15-0.37) | 0.0004 | 0.06 (0.02-0.16) | 0.25 (0.16-0.39) | 0.0003 | 0.05 (0.02-0.13) | 0.23 (0.14-0.36) | 0.0004 |
|  |  | **Scenario 6 (MixtureN + MixtureNV)** | | | **Scenario 7 (90 days, MedianSN + MedianNV)** | | | **Scenario 8 (PCR-positive only)** | | |
|  | **Level** | **Delta aHR* (CI)** | **Omicron aHR* (CI)** | **p-value** | **Delta aHR* (CI)** | **Omicron aHR* (CI)** | **p-value** | **Delta aHR* (CI)** | **Omicron aHR* (CI)** | **p-value** |
| **Age (years)** | <5 | 0.62 (0.36-1.06) | 0.47 (0.28-0.81) |  | 0.56 (0.30-1.06) | 0.38 (0.22-0.66) |  | 0.60 (0.30-1.19) | 0.71 (0.37-1.35) |  |
|  | 5-17 | 0.88 (0.62-1.26) | 0.76 (0.57-1.03) |  | 0.97 (0.64-1.46) | 0.71 (0.51-0.98) |  | 0.95 (0.61-1.47) | 0.88 (0.59-1.31) |  |
|  | 18-49 | ref | Ref |  | ref | ref |  | ref | ref |  |
|  | ≥50 | 1.60 (0.93-2.77) | 0.96 (0.58-1.56) | 0.85 | 1.13 (0.55-2.33) | 0.72 (0.43-1.21) | 0.62 | 1.60 (0.81-3.16) | 1.23 (0.63-2.41) | 0.80 |
| **Prior Infections** | 0 | ref | ref |  | ref | ref |  | ref | ref |  |
|  | 1 | 0.35 (0.24-0.52) | 0.72 (0.47-1.11) |  | 0.35 (0.23-0.53) | 0.74 (0.49-1.11) |  | 0.31 (0.20-0.49) | 0.81 (0.50-1.30) |  |
|  | ≥2 | 0.07 (0.03-0.16) | 0.29 (0.18-0.44) | 0.002 | 0.07 (0.02-0.30) | 0.30 (0.19-0.49) | 0.007 | 0.13 (0.05-0.36) | 0.48 (0.28-0.84) | 0.002 |

*P-values are for interaction and were calculated through likelihood ratio tests of models with and without interaction terms and are two-sided. All models use the Anderson-Gill extension of the proportional cox hazards model, accounting for clustering by participant and household. Number of infections defined as per scenarios in Table S1. *Calculated through the incorporation with an interaction term between the variable of interest and period with adjustment for prior infection status, age, household size and vaccination status.*

***Table S5. The frequency of SARS-CoV-2 symptoms reported by those with symptomatic infections***.

| **Symptom** | **Number reporting symptom (%)** |
| --- | --- |
| Fever | 15 (36.6) |
| Cough | 27 (65.9) |
| Headache | 16 (39.0) |
| Myalgia | 6 (14.6) |
| Anosmia | 5 (12.2) |
| Chest pain | 4 (9.8) |
| Shortness of breath | 0 (0) |
| Nasal congestion | 9 (22.0) |
| Sore throat | 6 (14.6) |
| Vomiting | 2 (4.9) |
| Diarrhoea | 0 (0) |

*Includes data from 41 symptomatic PCR-positive infection episodes.*

***Table S6. The univariate association between prior infection status, variant, age, household size and demographic/clinical variables with the odds of an infection being symptomatic.***

| **Variable** | **Level** | **Number of infections (%)*** | **Number of symptomatic infections (%)**** | **OR (95% CI)***** | **p-value** |
| --- | --- | --- | --- | --- | --- |
| Prior Infection | 0 | 94 (35.7) | 19 (20.2) | ref |  |
|  | 1 | 132 (50.2) | 17 (12.9) | 0.58 (0.28-1.19) |  |
|  | ≥2 | 31 (11.8) | 5 (16.1) | 0.76 (0.23-2.11) | 0.34 |
| Variant | Pre-Delta | 21 (8.0) | 4 (19.0) | ref |  |
|  | Delta | 111 (42.2) | 21 (18.9) | 0.99 (0.33-3.71) |  |
|  | Omicron | 125 (47.5) | 16 (12.8) | 0.62 (0.20-2.37) | 0.40 |
| Age (years) | 18-49 | 89 (33.8) | 19 (21.3) | ref |  |
|  | <5 | 25 (9.5) | 3 (12.0) | 0.50 (0.11-1.65) |  |
|  | 5-17 | 118 (44.9) | 14 (11.9) | 0.50 (0.23-1.05) |  |
|  | ≥50 | 25 (9.5) | 5 (20.0) | 0.92 (0.28-2.63) | 0.26 |
| Household Size | 5-7 | 100 (38.0) | 15 (15.0) | ref |  |
|  | 8-10 | 91 (34.6) | 17 (18.7) | 1.30 (0.61-2.81) |  |
|  | >10 | 66 (25.1) | 9 (13.6) | 0.89 (0.35-2.15) | 0.66 |
| HIV | Negative | 255 (97.0) | 41 (16.1) | ref |  |
|  | Positive | 2 (1.0) | 0 (0.0) | N/A | N/A |
| Employed | No | 213 (81.0) | 30 (14.1) | ref |  |
|  | Yes | 44 (16.7) | 11 (25.0) | 2.03 (0.90-4.37) | 0.09 |
| Steroids | No | 249 (94.7) | 40 (16.1) | ref |  |
|  | Yes | 8 (3.0) | 1 (12.5) | 0.75 (0.04-4.36) | 0.78 |
| History of Cancer | No | 256 (97.3) | 41 (16.0) | ref |  |
|  | Yes | 1 (0.3) | 0 (0.0) | N/A | N/A |
| Diabetes | No | 252 (95.8) | 38 (15.1) | ref |  |
|  | Yes | 5 (1.9) | 3 (60.0) | 8.45 (1.36-65.75) | 0.02 |
| Hypertension | No | 243 (92.4) | 37 (15.2) | ref |  |
|  | Yes | 14 (5.3) | 4 (28.6) | 2.23 (0.59-7.05) | 0.22 |
| Smoker | No | 253 (96.2) | 41 (16.2) | ref |  |
|  | Yes | 4 (1.5) | 0 (0.0) | N/A | N/A |
| Vaccinated | No | 233 (88.6) | 38 (16.3) | ref |  |
|  | Yes | 24 (9.1) | 3 (12.5) | 0.73 (0.17-2.26) | 0.62 |

*Only PCR-positive infections included. *% indicates the proportion of infections (n=257 with complete symptom data) within each variable level. Not all variables total 100% due to incomplete demographic/clinical data. **Calculated as the number of infections in each variable level that were symptomatic. ***Estimated from univariate logistic regression models with binary outcome (Symptomatic vs Asymptomatic). P-values calculated from likelihood ratio tests of nested models and are two-sided. N/A – indicates too few events for model fit, CI – Confidence Interval, ref – Reference Level.*

***Table S7. The univariate and multivariate associations between prior infection status, variant, and age with the odds of an infection being symptomatic.***

| **Variable** | **Level** | **Crude OR***  **(95% CI)** | **p-value (Crude)** | **Adjusted OR* (95% CI)** | **p-Value (Adjusted)** |
| --- | --- | --- | --- | --- | --- |
| Age | <5 | 0.91 (0.77-1.07) |  | 0.89 (0.75-1.05) |  |
|  | 5-17 | 0.91 (0.82-1.01) |  | 0.90 (0.81-0.99) |  |
|  | 18-49 | ref |  | ref |  |
|  | ≥50 | 0.99 (0.84-1.16) | 0.26 | 0.98 (0.83-1.15) | 0.16 |
| Prior Infection | 0 | ref |  | ref |  |
|  | 1 | 0.93 (0.84-1.02) |  | 0.92(0.83-1.02) |  |
|  | ≥2 | 0.96(0.83-1.11) | 0.33 | 0.96 (0.82-1.13) | 0.27 |
| Variant | Pre-Delta | ref |  | ref |  |
|  | Delta | 1.00 (0.84-1.19) |  | 1.01 (0.85-1.21) |  |
|  | Omicron | 0.94 (0.79-1.11) | 0.40 | 0.98 (0.82-1.17) | 0.75 |

*Only PCR-positive infections included. *Estimated from univariate logistic regression models with binary outcome (Symptomatic vs Asymptomatic). **Estimated from logistic regression modelling adjusting for age, prior infection status and variant period. P-values calculated from likelihood ratio tests of nested models and are two-sided. CI – Confidence Interval, ref – Reference Level.*

| **Variable** | **Level** | **Number exposed (%)*** | **Number infected (%)**** | **OR (95% CI)***** | **p-value** |
| --- | --- | --- | --- | --- | --- |
| Index Age (years) | <5 | 16 (3.6) | 1 (6.3) | ref |  |
|  | 5 – 17 | 214 (48.4) | 17 (7.9) | 1.25 (0.15-27.65) |  |
|  | 18-49 | 185 (41.9) | 16 (8.6) | 1.35 (0.16-29.86) |  |
|  | ≥50 | 27 (6.1) | 1 (3.7) | 0.61 (0.02-19.75) | 0.88 |
| Variant | Pre-Delta | 51 (11.5) | 1 (2.0) | ref |  |
|  | Delta | 224 (50.7) | 21 (9.4) | 6.08 (1.08-116.65) |  |
|  | Omicron | 167 (37.8) | 13 (7.8) | 4.68 (0.81-89.75) | 0.12 |
| Household Size (Median, IQR) |  | 7 (6-8) | 35 (7.9) | 0.78 (0.58-1.02) | 0.07 |
| Number of children in household (Median, IQR) |  | 4 (3-4) | 35 (7.9) | 0.92 (0.57-1.28) | 0.65 |
| Index Cycle Threshold (Median, IQR) |  | 31.41 (27.15-34.03) | 35 (7.9) | 1 (0.64-1.35) | 0.97 |
| Index Symptom Status | Asymptomatic | 361 (81.7) | 25 (6.9) | ref |  |
|  | Symptomatic | 59 (13.3) | 7 (11.9) | 1.95 (0.67-5.27) |  |
|  | Missing | 22 (4.9) | 3 (13.6) | 2.11 (0.43-7.92) | 0.31 |
| Serology | Negative | 173 (39.1) | 17 (9.8) | ref |  |
|  | Positive | 269 (60.9) | 18 (6.7) | 0.57 (0.24 – 1.25) | 0.16 |

***Table S8. Association between index participant factors and household factors and the odds of secondary transmission of SARS-CoV-2 in a single-index cluster (Secondary Attack Rate).***

*This includes just secondary transmission within one serial interval of the index case. *Calculated as the proportion of those exposed in each category. **Calculated as the number of exposed participants who subsequently tested SARS-CoV-2 positive. ***Calculated from a random effects logistic regression, with the variable of interest as a fixed effect and household as random effect. P-value calculated from likelihood ratio testing of nested models and are two-sided. CI – Confidence Interval, ref – Reference Level.*

***Table S9. Multivariate association between index SARS-CoV-2 serology and the odds of subsequent transmission of SARS-CoV-2 during a cluster after exposure to a single index (Secondary Attack Rate).***

| **Variable** | **Level** | **OR (95% CI)*** | **p-value** | **aOR (95% CI)**** | **p-value** |
| --- | --- | --- | --- | --- | --- |
| Serology | Negative | ref |  | ref |  |
|  | Positive | 0.57 (0.24 – 1.25) | 0.16 | 0.57 (0.23 – 1.30) | 0.23 |

*This includes just secondary transmission within one serial interval of the index case.*Calculated from a random effects logistic regression, with the variable of interest as a fixed effect and household as random effect.** Calculated from a random effects logistic regression, with the index SARS-CoV-2 serology and the contact’s serology as fixed effects and household as random effect. P-value calculated from likelihood ratio testing of nested models and are two-sided. CI – Confidence Interval, ref – Reference Level.*

***Table S10. Association between index participant factors and household factors and the odds of subsequent transmission of SARS-CoV-2 during a cluster after exposure to a single index (Household Cumulative Infection Risk).***

| **Variable** | **Level** | **Number exposed (%)*** | **Number infected (%)**** | **OR (95% CI)***** | **p-value** |
| --- | --- | --- | --- | --- | --- |
| Index Age (years) | <5 | 17 (3.6) | 2 (11.8) | ref |  |
|  | 5 – 17 | 228 (48.7) | 21 (9.2) | 0.73 (0.12-6.68) |  |
|  | 18-49 | 193 (41.2) | 19 (9.8) | 0.82 (0.13-7.50) |  |
|  | ≥50 | 28 (6.0) | 1 (3.6) | 0.29 (0.01-4.61) | 0.75 |
| Variant | Pre-Delta | 53 (11.3) | 3 (5.7) | ref |  |
|  | Delta | 236 (50.4) | 25 (10.6) | 2.28 (0.66-11.08) |  |
|  | Omicron | 177 (37.8) | 15 (8.5) | 1.59 (0.45-7.59) | 0.38 |
| Household Size (Median, IQR) |  | 7 (6-8) | 43 (9.2) | 0.86 (0.65-1.10) | 0.22 |
| Number of children in household (Median, IQR) |  | 4 (3-4) | 43 (9.2) | 0.99 (0.66-1.32) | 0.97 |
| Index Cycle Threshold (Median, IQR) |  | 31.41 (27.15-34.03) | 43 (9.2) | 0.97 (0.90-1.03) | 0.33 |
| Index Symptom Status | Asymptomatic | 380 (81.2) | 31 (8.2) | ref |  |
|  | Symptomatic | 62 (13.2) | 9 (14.5) | 2.14 (0.82-5.44) |  |
|  | Missing | 24 (5.1) | 3 (12.5) | 1.56 (0.32-5.68) | 0.26 |
| Serology | Negative | 183 (39.1) | 22 (12.0) | ref |  |
|  | Positive | 283 (60.5) | 21 (7.4) | 0.47 (0.21 – 0.99) | 0.05 |

*This includes all transmissions within a cluster after exposure to an index case (e.g., secondary, tertiary etc.). *Calculated as the proportion of those exposed in each category. **Calculated as the number of exposed participants who subsequently tested SARS-CoV-2 positive. ***Calculated from a random effects logistic regression, with the variable of interest as a fixed effect and household as random effect. P-value calculated from likelihood ratio testing of nested models and are two-sided. CI – Confidence Interval, ref – Reference Level.*

| **Variable** | **Level** | **OR (95% CI)*** | **p-value** | **aOR (95% CI)**** | **p-value** |
| --- | --- | --- | --- | --- | --- |
| Serology | Negative | ref |  | ref |  |
|  | Positive | 0.47 (0.21 – 0.99) | 0.05 | 0.57 (0.22 – 1.14) | 0.06 |

***Table S11. Multivariate association between index SARS-CoV-2 serology and the odds of subsequent transmission of SARS-CoV-2 during a cluster after exposure to a single index (Household Cumulative Infection Risk).***

*This includes all transmissions within a cluster after exposure to an index case (e.g., secondary, tertiary etc.) *Calculated from a random effects logistic regression, with the variable of interest as a fixed effect and household as random effect.** Calculated from a random effects logistic regression, with the index SARS-CoV-2 serology and the contact’s serology as fixed effects and household as random effect. P-value calculated from likelihood ratio testing of nested models and are two-sided. CI – Confidence Interval, ref – Reference Level.*

## ***Table S12. Summary of PCR-negative scenarios for sensitivity analyses***

| Scenario | Definition |
| --- | --- |
| Base | No re-infection within 28 days, PCR-negative infection defined by a median-fold change in Spike and Nucleocapsid antibody levels in non-vaccinated participants and a median-fold change in Nucleocapsid antibody levels in vaccinated participants. |
| 1 | No re-infection within 28 days, PCR-negative infection defined by a IQR1-fold change in Spike and Nucleocapsid antibody levels in non-vaccinated participants and a median-fold change in Nucleocapsid antibody levels in vaccinated participants. |
| 2 | No re-infection within 28 days, PCR-negative infection defined by a IQR1-fold change in Spike and Nucleocapsid antibody levels in non-vaccinated participants and a IQR1-fold change in Nucleocapsid antibody levels in vaccinated participants. |
| 3 | No re-infection within 28 days, PCR-negative infection defined by a median-fold change in Spike antibody levels in non-vaccinated participants and a median-fold change in Nucleocapsid antibody levels in vaccinated participants. |
| 4 | No re-infection within 28 days, PCR-negative infection defined by a median-fold change in Nucleocapsid antibody levels in non-vaccinated participants and a median-fold change in Nucleocapsid antibody levels in vaccinated participants. |
| 5 | No re-infection within 28 days, PCR-negative infections defined by a threshold value in Spike in non-vaccinated participants and Nucleocapsid in vaccinated participants. Threshold estimated with mixture modelling (below, Equation 1) |
| 6 | No re-infection within 28 days, PCR-negative infections defined by a threshold value in Nucleocapsid in non-vaccinated participants and Nucleocapsid in vaccinated participants. Threshold estimated with mixture modelling (below, Equation 1) |
| 7 | No re-infection within 90 days, PCR-negative infection defined by a median-fold change in Spike and Nucleocapsid antibody levels in non-vaccinated participants and a median-fold change in Nucleocapsid antibody levels in vaccinated participants. |
| 8 | No re-infection with 28 days, PCR-negative infections not included. |

##

## ***Determining the seroconversion threshold through mixture modelling***

To determine the threshold value for seroconversion, we assume that the fold-rise between two-time points (*x*) for an antigenic protein (*p*) follows a mixture normal distribution;

$$t_{p,x} \sim pN\left( \mu_{-},\sigma_{-} \right)+\left( 1-p \right)N(\mu_{+},\sigma_{+})$$

where 0 ≤ *p* ≤ 1, $\mu_{-}$ and $\sigma_{-}$ represents the mean and standard deviation for the population who have not seroconverted and , $\mu_{+}$ and $\sigma_{+}$ represents the mean and standard deviation for the population who have seroconverted. The normal distribution representing the population not seroconverted is censored at 1, to represent the lower threshold value of the SARS-CoV-2 binding antibody ELISA. The threshold value for seroconversion is the local minimum between $\mu_{-}$and $\mu_{+}$We fit a mixture normal distributions to all the spike and all the ncp data separately. To assess the influence of pre-season titre on seroconversion threshold, we also stratify these data by their pre-season titre {<1, 1-32, 32+} and fit separate mixture normal distributions to each level.

To fit the five-parameter $\{p, \mu_{-},\sigma_{-},\mu_{+},\sigma_{+}\}$ mixture normal distributions to each dataset, we use the *mixfit* function in the *janitor* package in R.

Estimated dates for PCR-negative infections to include in incidence modelling were derived by random, weighted-sampling based on distributions defined by SARS-CoV-2 waves in The Gambia during a given time period (Figure S1).

## ***Figure S1. Imputed dates of PCR-negative infections for scenarios B, and 1-7.*** *For each scenario the imputed PCR-negative infections dates (bottom plot) are compared against the epidemic curve of The Gambia during TransVir follow-up (top plot).*

***Base***

***
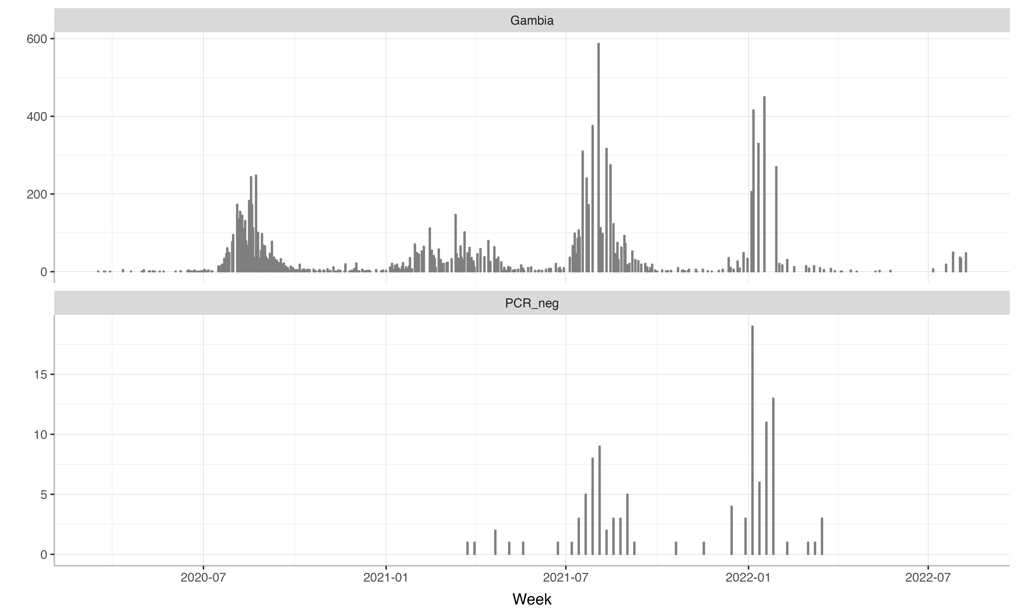
***

***Scenario 1***

***
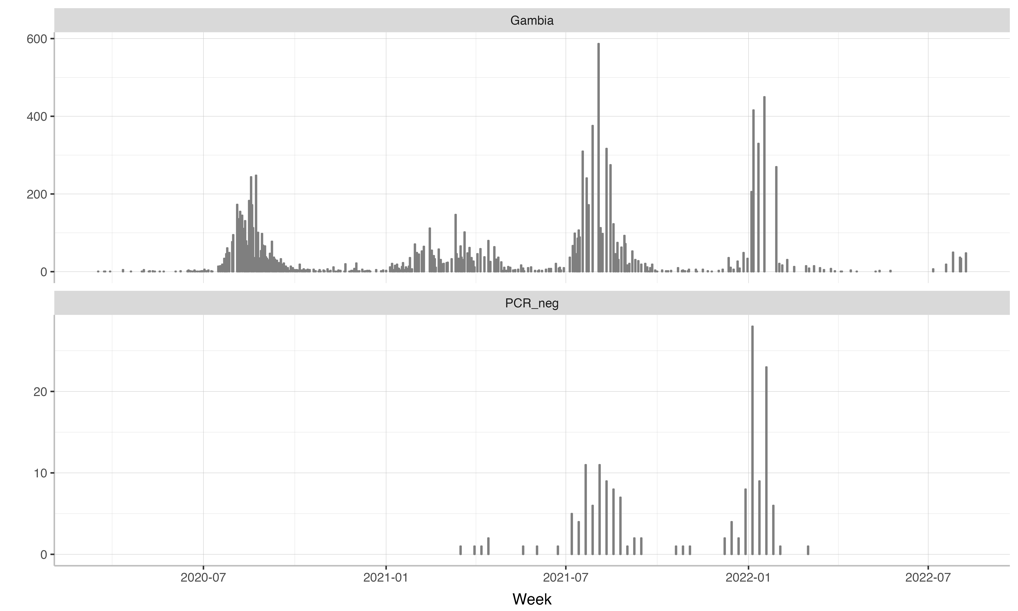
***

***Scenario 2***

***
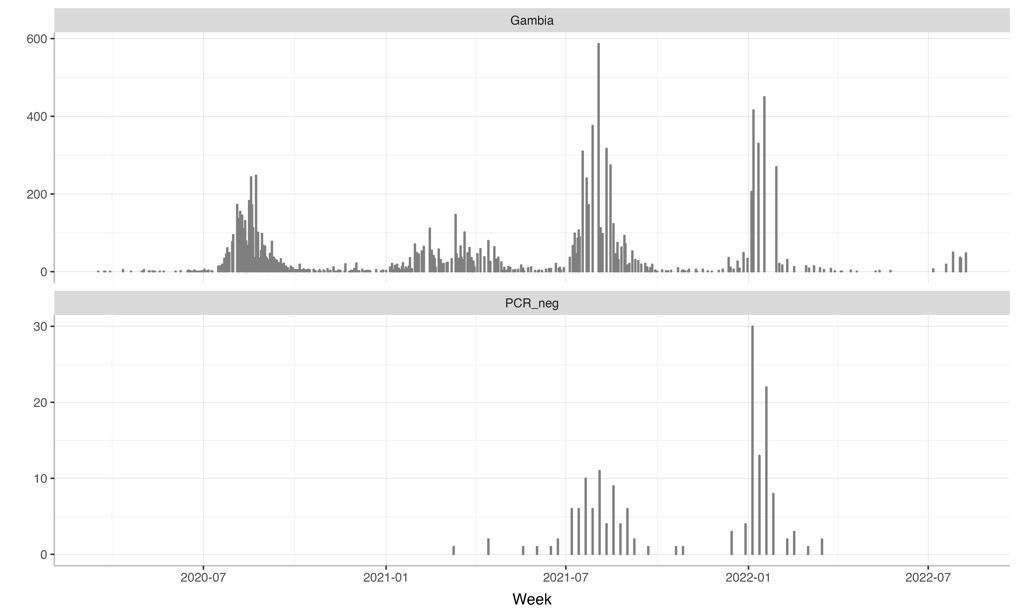
***

***Scenario 3***

***
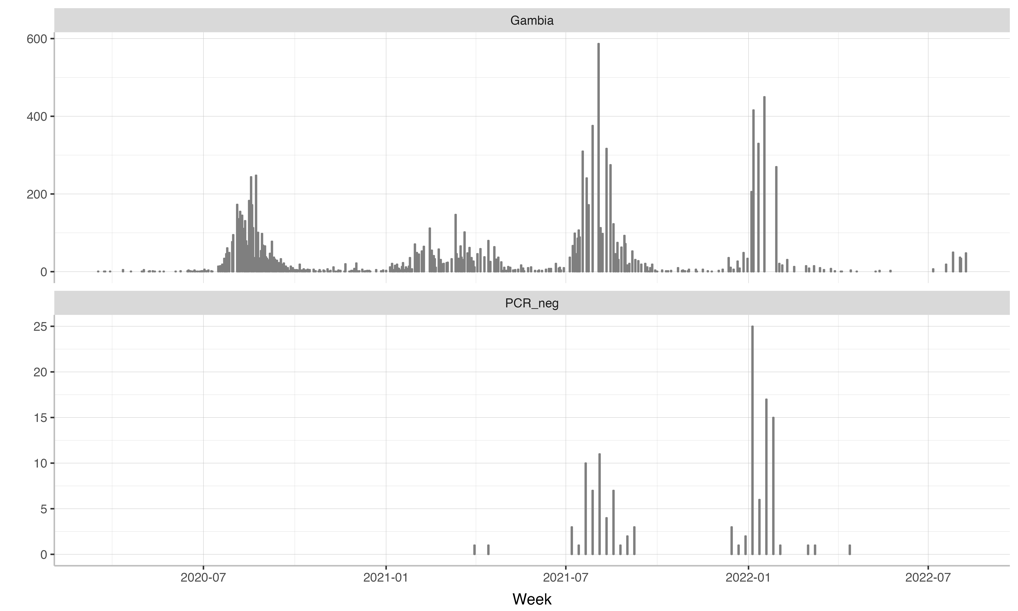
***

***Scenario 4***

***
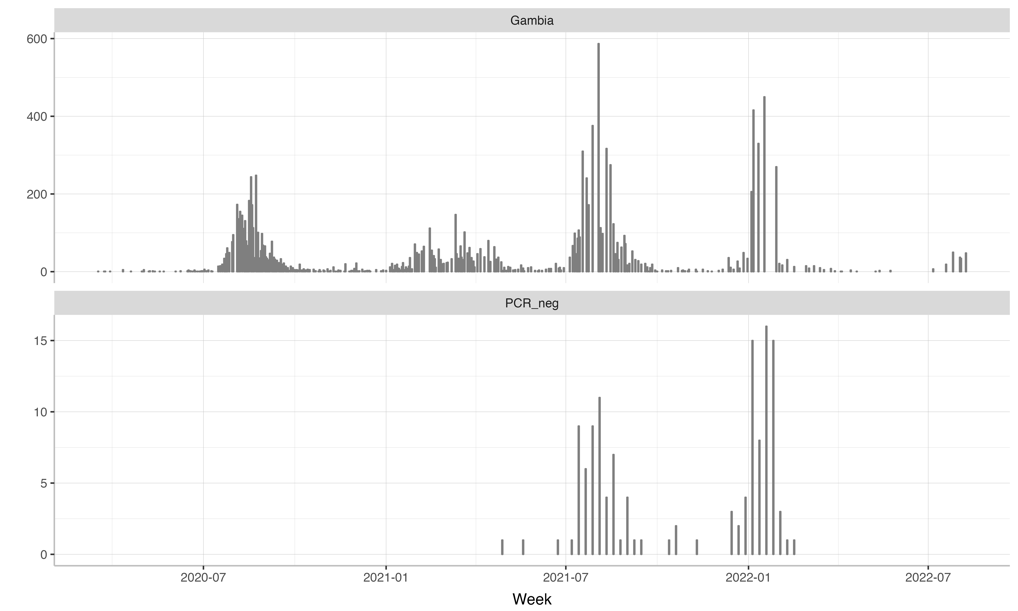
***

***
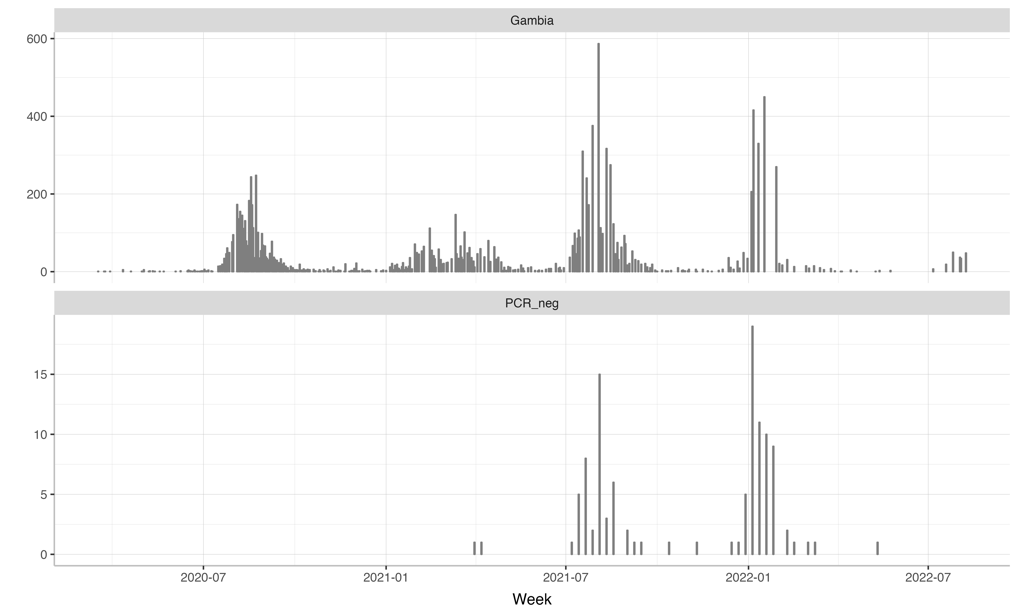
Scenario 5***

***Scenario 6***

***
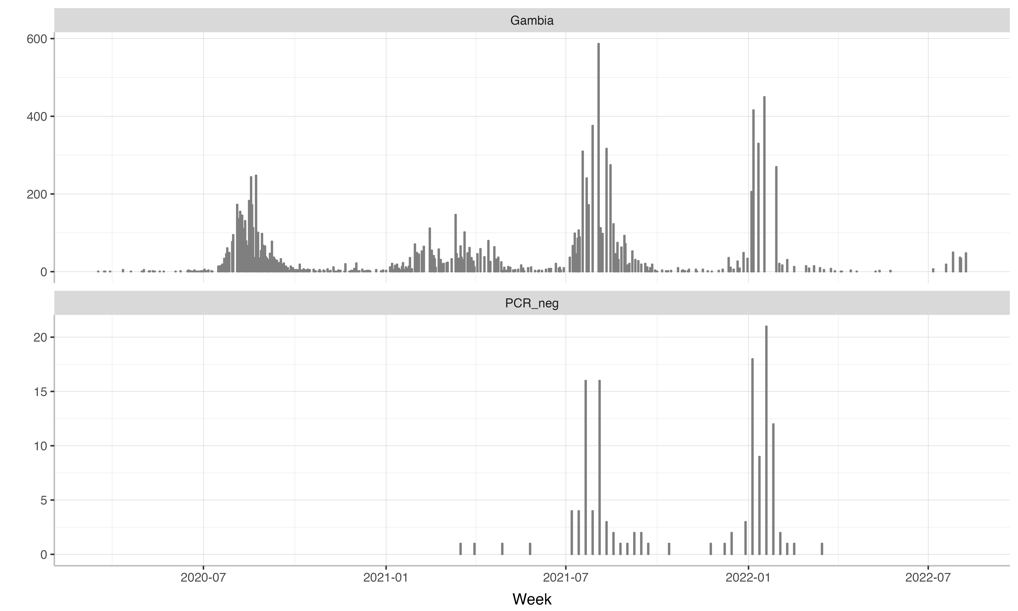
***

***Scenario 7***

***
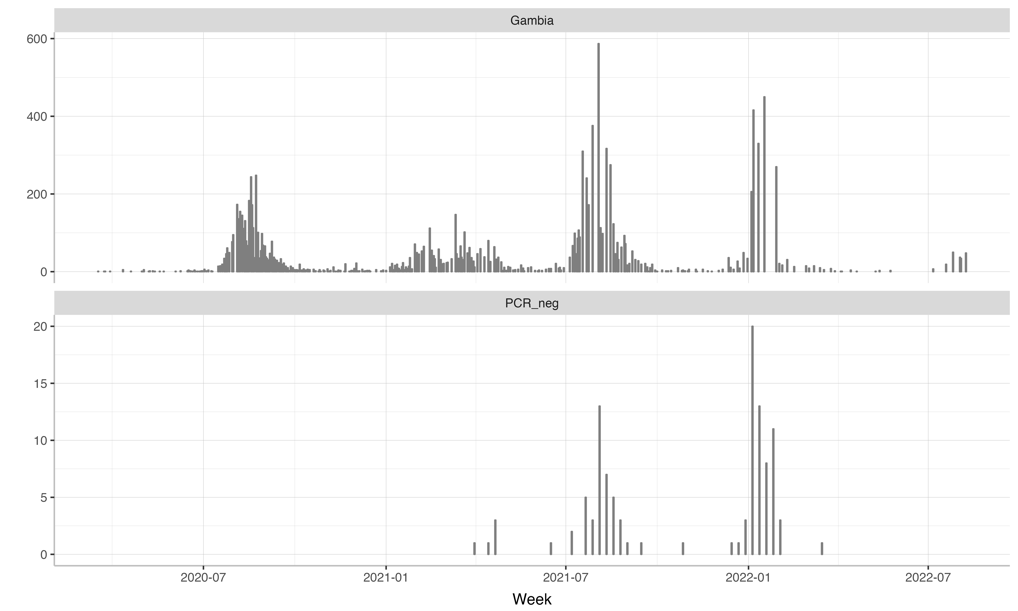
***
