# Supplementary material for: High SARS-CoV-2 incidence and asymptomatic fraction during Delta and Omicron BA.1 waves in The Gambia
Source: Nat Commun. 2024 May 7;15:3814. doi: 10.1038/s41467-024-48098-3 (PMC11076623; doi:10.1038/s41467-024-48098-3)
Supplement: Supplementary file 3 — Reporting Summary [file 41467_2024_48098_MOESM3_ESM.pdf]

nature portfolio

Corresponding author(s): Thushan de Silva

Last updated by author(s): Apr 10, 2024

Reporting Summary

Nature Portfolio wishes to improve the reproducibility of the work that we publish. This form provides structure for consistency and transparency in reporting. For further information on Nature Portfolio policies, see our Editorial Policies and the Editorial Policy Checklist.

Statistics

For all statistical analyses, confirm that the following items are present in the figure legend, table legend, main text, or Methods section.

n/a	Confirmed	
☐	☒	The exact sample size (*n*) for each experimental group/condition, given as a discrete number and unit of measurement
☐	☒	A statement on whether measurements were taken from distinct samples or whether the same sample was measured repeatedly
☐	☒	The statistical test(s) used AND whether they are one- or two-sided<br>*Only common tests should be described solely by name; describe more complex techniques in the Methods section.*
☐	☒	A description of all covariates tested
☐	☒	A description of any assumptions or corrections, such as tests of normality and adjustment for multiple comparisons
☐	☒	A full description of the statistical parameters including central tendency (e.g. means) or other basic estimates (e.g. regression coefficient) AND variation (e.g. standard deviation) or associated estimates of uncertainty (e.g. confidence intervals)
☐	☒	For null hypothesis testing, the test statistic (e.g. *F*, *t*, *r*) with confidence intervals, effect sizes, degrees of freedom and *P* value noted<br>*Give P values as exact values whenever suitable.*
☒	☐	For Bayesian analysis, information on the choice of priors and Markov chain Monte Carlo settings
☒	☐	For hierarchical and complex designs, identification of the appropriate level for tests and full reporting of outcomes
☒	☐	Estimates of effect sizes (e.g. Cohen's *d*, Pearson's *r*), indicating how they were calculated

Our web collection on statistics for biologists contains articles on many of the points above.

Software and code

Policy information about availability of computer code

Data collection	Clinical/demographic data was collected by field workers and inputted into REDCap
Data analysis	Data reformatting, manipulation and analysis was performed in R using the packages readxl (v1.4.3), tidyverse (v2.0), ggplot2 (v3.4.2), survival (v3.5-5), lubridate (v1.9.3), survminer (v.0.4.9), broom (v1.0.5), popEpi (v.0.4.11), and EpiR (v.2.0.62).

For manuscripts utilizing custom algorithms or software that are central to the research but not yet described in published literature, software must be made available to editors and reviewers. We strongly encourage code deposition in a community repository (e.g. GitHub). See the Nature Portfolio guidelines for submitting code & software for further information.

Data

Policy information about availability of data

All manuscripts must include a data availability statement. This statement should provide the following information, where applicable:

- Accession codes, unique identifiers, or web links for publicly available datasets
- A description of any restrictions on data availability
- For clinical datasets or third party data, please ensure that the statement adheres to our policy

Aggregate data to reproduce figures are available online at https://github.com/Rhys-wenlock/Transvir_SCV2_incidence. Individual-level data cannot by publicly shared due to ethical restrictions and the potential for identifying included individuals. To request individual participant data access, please contact the corresponding author T.d.S (t.desilva@sheffield.ac.uk) who will respond within 1 month of request. Upon approval, data can be made available through a data

sharing agreement. All data and code used to perform the analyses can be found at https://github.com/Rhys-wenlock/Transvir_SCV2_incidence and at the DOI:10.5281/zenodo.10955388.

Research involving human participants, their data, or biological material

Policy information about studies with human participants or human data. See also policy information about sex, gender (identity/presentation), and sexual orientation and race, ethnicity and racism.

Reporting on sex and gender	57.6% of participants were female. No further analysis based on sex/gender was performed.
Reporting on race, ethnicity, or other socially relevant groupings	All participants were Gambian. Further data on race, ethnicity or other socially relevant groupings were not collected.
Population characteristics	349 participants from 52 households in the West Coast Region and Kanifing Municipal Area of The Gambia were recruited, with a median of 6 participants per household. 41 participants were children under 5 years old (11.7%), 153 were 5-17 years old (43.8%), 130 were 18-49 years old (37.2%) and 25 were ≥50 years old (7.2%); with 201 (57.6%) female participants. Recruitment commenced during the 2nd SARS-CoV-2 wave in The Gambia (Fig. 1), with V1 visits completed prior to the Delta (3rd) wave and most V2 visits completed prior to the Omicron BA.1 (4th) wave. 56% of participants at recruitment were seropositive to Spike antigen, with 47% positive to Nucleocapsid antigen. No participants had been vaccinated against SARS-CoV-2 at baseline.
Recruitment	The study was conducted at two urban sites, the West Coast Region and Kanifing Municipality of the Gambia. Both sites had households that had previously participated in studies conducted at the Medical Research Council Unit The Gambia at The London School of Hygiene & Tropical Medicine (MRCG). These sites were selected due to established relationships with these communities that facilitated participant recruitment during the COVID-19 pandemic. All households that had previously participated in research conducted by MRCG within these communities were approached for study sensitisation and gauge interest in participating in the study. Households expressing interest in joining the study were invited to attend MRCG for informed consent and screening. All households with ≥5 consenting members including at least one adult and one child were eligible. Written (or thumb-printed) informed consent was obtained from all adult participants, assent was obtained from children aged 12-18 years, and parents or guardians provided consent for children <12 years old.<br><br>By approaching selected households based on prior community engagement may have skewed the age and/or household size distribution, which in turn could have had an impact on the generalisability of our data to the wider population. However, the median age in our study is the same as the general population in The Gambia (17 years). Health seeking behaviour may also have been different in our study participants compared to the wider community. As we did routine sampling for SARS-CoV-2 RT-PCR rather than rely on symptomatic illness alone, we would expect this to have minimal impact on our findings. However, as our participant selection was not a random selection from the chosen community, there could be unknown bias in social contact behaviour which could impact risk of acquiring SARS-CoV-2.
Ethics oversight	The TransVIR study (Transmission of Respiratory Viruses in Household in The Gambia), was approved by the joint Gambia Government and MRCG ethics committee, and the London School of Hygiene and Tropical Medicine ethics committee (ID 22556).

Note that full information on the approval of the study protocol must also be provided in the manuscript.

Field-specific reporting

Please select the one below that is the best fit for your research. If you are not sure, read the appropriate sections before making your selection.

☒ Life sciences ☐ Behavioural & social sciences ☐ Ecological, evolutionary & environmental sciences

For a reference copy of the document with all sections, see nature.com/documents/nr-reporting-summary-flat.pdf

Life sciences study design

All studies must disclose on these points even when the disclosure is negative.

Sample size	The study design aims to provide sufficient data to capture within household dynamics between different groups, as well as a precise estimate of infection and re-infection incidence. We aimed to recruit between 50 to 70 households, aiming to recruit a maximum of 500 participants (assuming a median household size of 7). Given the uncertainty on future incidence, and on the prevalence of variables of interest (e.g., seroprevalence), formal power calculations were difficult. Three-hundred-and-fifty participants (minimum 50 households recruited with median household size of 7) would be able to estimate an incidence risk with a 12% absolute precision (+/- 6%), allowing for a 1.3 design effect household clustering) and 95% confidence. With 40% of the population estimated to be children, 350 participants would be expected to provide an 80% power (at alpha=0.05) to detect a 20% higher absolute incidence risk in adults.
Data exclusions	No data exclusions
Replication	Not applicable - this was an epidemiological observational study and not an experimental one.
Randomization	Allocation to groups was not not random, study participants were defined by demographic variables (e.g., age, household size), their prior

Randomization	infection history (based upon a combination of prior serological testing and RT-PCR history), vaccination status, or the time of follow-up (variant period).
Blinding	Not applicable - this was an observational study and there was no intervention in this study.

Reporting for specific materials, systems and methods

We require information from authors about some types of materials, experimental systems and methods used in many studies. Here, indicate whether each material, system or method listed is relevant to your study. If you are not sure if a list item applies to your research, read the appropriate section before selecting a response.

Materials & experimental systems

n/a	Involved in the study
☐	☒ Antibodies
☒	☐ Eukaryotic cell lines
☒	☐ Palaeontology and archaeology
☐	☒ Animals and other organisms
☐	☒ Clinical data
☒	☐ Dual use research of concern
☒	☐ Plants

Methods

n/a	Involved in the study
☒	☐ ChIP-seq
☒	☐ Flow cytometry
☒	☐ MRI-based neuroimaging

Antibodies

Antibodies used	In-house anti-Spike (S) and anti-Nucleocapsid (N) ELISAs were used to determine SARS-CoV-2 serostatus. A goat anti-human IgG antibody conjugated to horse radish peroxidase (Invitrogen, 62-8420) was used at 1:500 dilution in both anti-S and -N IgG ELISA assays as the secondary antibody.<br><br>A standard curve calibrated to the WHO International Standard for anti-SARS-CoV-2 Immunoglobulin (cat no. NIBSC 20/136) was used to quantify S and N antibody titres.
Validation	SARS-CoV-2 spike- (S) and N-specific immunoglobulin G (IgG) was measured using a previously described in-house enzyme-linked immunosorbent assay (ELISA), shown to have 99.5% sensitivity and 98.8% specificity for anti-S IgG, and 99.5% sensitivity and 84.1% specificity for anti-N IgG as described in Colton H, Hodgson D, Hornsby H, et al. Risk factors for SARS-CoV-2 seroprevalence following the first pandemic wave in UK healthcare workers in a large NHS Foundation Trust. Wellcome Open Res. 2022;6:220. Published 2022 Jun 10. doi:10.12688/wellcomeopenres.17143.3

Animals and other research organisms

Policy information about studies involving animals; ARRIVE guidelines recommended for reporting animal research, and Sex and Gender in Research

Laboratory animals	*For laboratory animals, report species, strain and age OR state that the study did not involve laboratory animals.*
Wild animals	*Provide details on animals observed in or captured in the field; report species and age where possible. Describe how animals were caught and transported and what happened to captive animals after the study (if killed, explain why and describe method; if released, say where and when) OR state that the study did not involve wild animals.*
Reporting on sex	*Indicate if findings apply to only one sex; describe whether sex was considered in study design, methods used for assigning sex. Provide data disaggregated for sex where this information has been collected in the source data as appropriate; provide overall numbers in this Reporting Summary. Please state if this information has not been collected. Report sex-based analyses where performed, justify reasons for lack of sex-based analysis.*
Field-collected samples	*For laboratory work with field-collected samples, describe all relevant parameters such as housing, maintenance, temperature, photoperiod and end-of-experiment protocol OR state that the study did not involve samples collected from the field.*
Ethics oversight	*Identify the organization(s) that approved or provided guidance on the study protocol, OR state that no ethical approval or guidance was required and explain why not.*

Note that full information on the approval of the study protocol must also be provided in the manuscript.

Clinical data

Policy information about clinical studies

All manuscripts should comply with the ICMJE guidelines for publication of clinical research and a completed CONSORT checklist must be included with all submissions.

Clinical trial registration	Registered at clinicaltrials.gov with the ID: NCT05952336
Study protocol	Study protocol included in supplementary materials and on clinicaltrials.gov website.
Data collection	Demographic (e.g., age, sex, household factors) and clinical data (e.g., vaccination status, co-morbidities, relevant medications) were collected at clinic visits conducted at baseline, 6-months and 12-months alongside venous blood sampling for serological testing (anti-Spike and anti-Nucleocapsid). Weekly home visits were then conducted by field workers for the duration of the study with an assessment of influenza-like-symptoms in the week preceeding the visit, and the collection of a combination throat and nasal swab for SARS-CoV-2 RT-PCR testing. Participants were informed that if they developed influenza-like-symptoms they should contact the research team for an unscheduled visit, with rapid turnaround of SARS-CoV-2 RT-PCR results. All data was inputted into a REDCap database, with quality control performed by a dedicated database manager.
Outcomes	1. Incidence of SARS-CoV-2 infection as determined by molecular (e.g. PCR) and serological testing and the association between infection incidence and demographic, clinical and SARS-CoV-2 related variables<br>2. Secondary attack rate and household cumulative infection rate with SARS-CoV-2, and associations with demographic, clinical and SARS-CoV-2 related variables<br>3. The proportion of infections that are symptomatic, and risk factors for symptomatic infection.

Plants

Seed stocks	*Report on the source of all seed stocks or other plant material used. If applicable, state the seed stock centre and catalogue number. If plant specimens were collected from the field, describe the collection location, date and sampling procedures.*
Novel plant genotypes	*Describe the methods by which all novel plant genotypes were produced. This includes those generated by transgenic approaches, gene editing, chemical/radiation-based mutagenesis and hybridization. For transgenic lines, describe the transformation method, the number of independent lines analyzed and the generation upon which experiments were performed. For gene-edited lines, describe the editor used, the endogenous sequence targeted for editing, the targeting guide RNA sequence (if applicable) and how the editor was applied.*
Authentication	*Describe any authentication procedures for each seed stock used or novel genotype generated. Describe any experiments used to assess the effect of a mutation and, where applicable, how potential secondary effects (e.g. second site T-DNA insertions, mosiacism, off-target gene editing) were examined.*
